# Increased risk of chikungunya infection in travellers to Thailand during ongoing outbreak in tourist areas: cases imported to Europe and the Middle East, early 2019

**DOI:** 10.2807/1560-7917.ES.2019.24.10.1900146

**Published:** 2019-03-07

**Authors:** Emilie Javelle, Simin-Aysel Florescu, Hilmir Asgeirsson, Shilan Jmor, Gilles Eperon, Eyal Leshem, Johannes Blum, Israel Molina, Vanessa Field, Nancy Pietroski, Carole Eldin, Victoria Johnston, Ioana Ani Cotar, Corneliu Popescu, Davidson H Hamer, Philippe Gautret

**Affiliations:** 1 Laveran Military Teaching Hospital, Marseille, France; 2 IHU-Méditerranée Infection, Marseille, France; 3 Aix Marseille Université, IRD, AP-HM, SSA, VITROME, Marseille, France; 4 Carol Davila University of Medicine and Pharmacy, Clinical Hospital of Infectious and Tropical Diseases ‘Dr. Victor Babes’, Bucharest, Romania; 5 Department of Infectious Diseases, Karolinska University Hospital, Stockholm, Sweden; 6 Unit of Infectious Diseases, Department of Medicine Huddinge, Karolinska Institutet, Stockholm, Sweden; 7 Hospital for Tropical Diseases, London, United Kingdom; 8 Division of Tropical and Humanitarian Medicine, Department of Primary Care, Geneva University Hospitals (HUG), Geneva, Switzerland; 9 The Center for Travel and Tropical Medicine, Sheba Medical Center and Sackler Faculty of Medicine, Tel Aviv University, Israel; 10 FMH Innere Medizin und Tropen- und Reisemedizin, Schweizerisches Tropen- und Public Health Institut, Basel, Switzerland; 11 Department of Infectious Diseases, Hospital Universitari Vall d’Hebron, PROSICS Barcelona, Universitat Autònoma de Barcelona, Barcelona, Spain; 12 Chair of the Tracking and Communications Working Group, GeoSentinel, London, United Kingdom.; 13 International Society of Travel Medicine, Dunwoody, United States; 14 ‘Cantacuzino’ National Medico-Military Institute for Research and Development, Bucharest, Romania; 15 Department of Global Health, Boston University School of Public Health, Boston, United States; 16 Section of Infectious Diseases, Department of Medicine, Boston Medical Center, Boston, United States

**Keywords:** chikungunya, Thailand, outbreak, travellers, importation

## Abstract

We report nine travellers with confirmed chikungunya virus infection, returning from tourist areas of Thailand to Sweden, Switzerland, the United Kingdom, Romania, Israel and France, diagnosed in January and February 2019. These sentinel tourists support the intensification of chikungunya virus circulation in Thailand and highlight the potential for importation to areas at risk of local transmission.

Since the start of 2019, the EuroTravNet/GeoSentinel and TropNet data collection networks for the surveillance of travel-related morbidity have identified nine patients with chikungunya virus (CHIKV) infection imported from Thailand to Sweden, Switzerland, the United Kingdom (UK), Romania, Israel and France. In comparison, the last CHIKV infection reported to EuroTravNet/GeoSentinel in travellers from Thailand was a suspected case in Romania in January 2018. Only three other cases were reported to this network during the past 3 years from Thailand, and none in travellers returning to Europe. Here, we present the clinical and travel data of eight travellers notified to EuroTravNet/GeoSentinel and one notified to TropNet with confirmed chikungunya disease imported from Thailand within 2 months. 

## Characteristics of travellers returning from Thailand with chikungunya virus infection

The average length of stay of the nine travellers in Thailand was 22 days (range: 10–35 days) and the average delay between arrival in Thailand and onset of chikungunya symptoms was 16 days (range: 5–22 days). Places visited by the travellers in the period before chikungunya onset are shown in the Figure.

**Figure:**
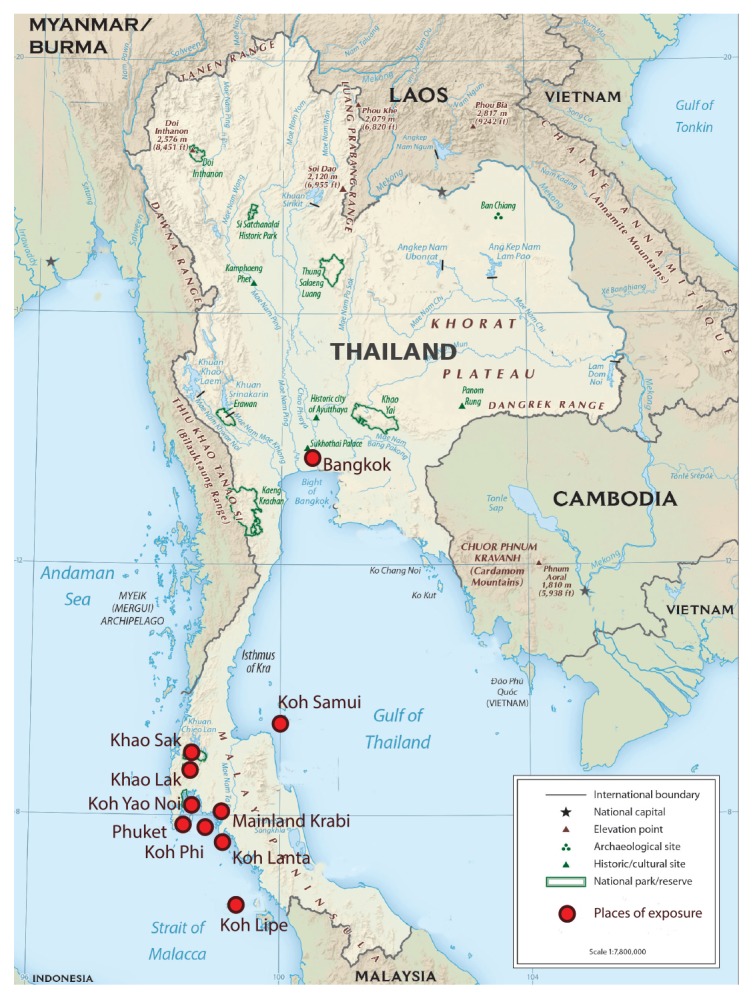
Places of exposure visited by travellers before chikungunya virus infection, Thailand, January–February 2019 (n = 9)

Source: Map from Wikimedia Commons, the free media repository: https://commons.wikimedia.org/wiki/File:Thailand_Physiography.jpg

The patients’ median age was 37 years (range: 14–73 years), and seven of the nine were female. Underlying conditions were reported in three patients (Table). Acute symptoms included fever and joints pains in all patients, seven had a rash, five reported headache, two had lymphadenitis, five developed oedema or joint swelling (acute thrombophlebitis was excluded in Case 3). Case 8 experienced malaise, fever and loss of consciousness and subsequent head trauma because of the fall. Three patients were hospitalised in Thailand (Cases 1, 3 and 8) and one after returning to their home country (Case 4); the remaining five were outpatients. All nine cases were confirmed after their return at the laboratory of the reporting site as detailed in the Table. Alternative diagnoses including arboviral infection with dengue and Zika viruses as well as malaria were ruled out at the time of chikungunya diagnosis using smear tests, dengue virus NS1-antigen, RT-PCR and/or serological tests depending on the delay since onset of symptoms and using tests referenced at the different diagnostic laboratories mentioned in the Table. 

**Table:** Overview of nine confirmed chikungunya cases among travellers returning from Thailand, by date of onset, January–February 2019 (n = 9)

Case	Reporting country	Places visited	Period of exposure	Age^a^	Underlying conditions^b^	Date of onset	Clinical acute symptoms	Outcome	Recovery status as at 22 Feb 2019	Diagnosis method (positive tests)
1	Sweden	Koh Lanta	27 Oct–28 Nov 2018	75 years	Yes	19 Nov 2018	Fever and arthralgia	Persistent arthralgia and arthritis in fingers, wrists and right foot; pains in upper arms; difficulties with walking and activities of daily life	No (chronic chikungunya)	Anti-CHIKV IgM ELISA (InBios, Seattle, US) and IgG (in-house IF) in week 6 at Karolinska University Hospital, Stockholm
2	Sweden	Koh Lanta	16 Dec 2018–2 Jan 2019	30 years	None	4 Jan 2019	Fever, lymphadenitis, arthralgia, myalgia, headache and diffuse rash	Persistent arthralgia, difficulties walking long distances, special regime of half-time work	No	Anti-CHIKV IgM ELISA (InBios, Seattle, US) and IgG (in-house IF) on day 14 at Karolinska University Hospital Stockholm
3	Switzerland	Bangkok, Phuket, Koh Yao Noi, Kho Phi, Chang Mai from 8 Jan 2019	18 Dec 2018–12 Jan 2019	50 years	Yes	5 Jan 2019	4-day fever, polyarthralgia, myalgia, rash and asthenia	Persistent incapacitating joint pain and oedema in lower limbs	No	Anti-CHIKV IgM and IgG ELISA (Euroimmun, Lübeck, Germany) on day 18 at Geneva University Hospital
4	Romania	Phuket and Kata Beach	3–13 Jan 2019	15 years	None	12 Jan 2019	2-day fever, arthralgia mainly involving the hands, axillary lymphadenitis and rash on the upper and lower extremities	Recovered	Yes	CHIKV RT-PCR [1] on day 2, anti-CHIKV IgM and IgG ELISA (Euroimmun, Lübeck, Germany) on day 19 at Cantacuzino Institute, Bucharest
5	United Kingdom	Bangkok, Khao Lak, Khao Sok, mainland Krabi and Koh Lipe	25 Dec 2018–15 Jan 2019	25 years	None	13 Jan 2019	Fever, ankle pain and mild generalised headache	Persistent arthralgia	No	CHIKV RT-PCR [1] and anti-CHIKV IgM ELISA (Euroimmun, Lübeck, Germany) on day 4 at Hospital for Tropical Diseases, London
6	United Kingdom	Bangkok, Khao Lak, Khao Sok, mainland Krabi and Koh Lipe	25 Dec 2018–15 Jan 2019	35 years	None	14 Jan 2019	Fever, joint pain in ankles and back, generalised headache, transient rash on chest and widespread rash on day 8	Persistent arthralgia	No	CHIKV RT-PCR on day 3 [1] at Hospital for Tropical Diseases, London
7	Israel	Koh Samui	13 Jan–3 Feb 2019	20 years	None	18 Jan 2019	Fever, rash and join pain	Persistent arthralgia	No	Anti-CHIKV IgM and IgG ELISA (Euroimmun, Lübeck, Germany) on day 24 at Sheba Medical Center, Tel Aviv
8	France	Bangkok, Koh Lanta and Koh Samui	15–31 Jan 2019	35 years	Yes	25 Jan 2019	3-day fever, headache, nausea, vomiting, malaise, rash and joint pain	Acute left ankle synovitis requiring crutches to walk on day 8	Yes	CHIKV RT-PCR [1] on day 7, anti-CHIKV IgM ELISA (Euroimmun, Lübeck, Germany) on day 10 at CNR IHU-Mediterranean Infection, Marseille
9	Switzerland	Phuket	9 Jan–13 Feb 2019	60 years	None	25 Jan 2019	3-day fever, chills, headache, myalgia, arthralgia, abdominal pain, 3-day itching generalised rash, dysgeusia	Swelling joint pain in both feet and knees, neck pains and irritation of the oesophagus	No	Anti-CHIKV IgM and IgG, ELISA (Euroimmun, Lübeck, Germany) on day 24 at Schweizerisches Tropen-Institut, Basel

CHIKV: chikungunya virus; IF: immunofluorescence; IgG: immunoglobulin G; US: United States.

^a^ Ages are rounded to the nearest five-year age group.

^b^ Underlying conditions that occurred: hypothyroidism (taking levothyroxin), hypertension (treated) and pancreas resection; familial hypercholesterolaemia; beta thalassaemia and carpal tunnel syndrome.

Eight of the nine CHIKV-infected travellers received non-steroidal anti-inflammatory drugs (NSAID) for persistent arthralgia. Case 9 was treated with intravenous corticosteroids at the acute stage in Thailand. Only two travellers completely recovered within 3 weeks (Case 4 and Case 8). In contrast, the woman in her 70s (Case 1) developed post-chikungunya chronic rheumatism (lasting more than 3 months). She is heavily impaired in her activities of daily life and has been referred to rheumatology for evaluation.

## Simultaneous case of dengue infection imported from Thailand

A teenage sibling travelling with Case 4 developed fever 4 days before leaving Thailand, followed by a petechial rash, mild arthralgia, nausea, vomiting and mild axillary lymphadenitis. On the day after return to their country of residence, NS1 antigen was positive for dengue virus and RT-PCR for dengue-1 virus was positive in urine while RT-PCR and serology for CHIKV-infection were negative in serum [1]. Their parents travelling with them have remained asymptomatic.

## Discussion

Beginning in October 2018, the Thai Ministry of Public Health reported a major increase in the number of chikungunya cases in the country. In November alone, 1,132 cases were reported, and by 10 December 2018, this had risen to 2,143 cases, compared with a total of 10 cases in 2017 [2]. From 1 January to 10 February 2019, 1,652 patients from 17 provinces were reported; mostly affecting Phuket, Pattani, Songkhla, Narathiwat and Phang Nga provinces, with the highest morbidity rate in the southern region [3].

We suspect that all infections in our traveller case series were acquired in southern Thailand. For people who develop symptomatic chikungunya illness, the incubation period is typically 4–7 days (range: 1–12 days) [4]. Bangkok was the point of entry (airport) with a short initial stop for Cases 3, 5, 6 and 8 who developed first symptoms, respectively, 18, 19, 20 and 11 days after their arrival. Case 3 visited Chang Mai in the north of Thailand after the onset of chikungunya symptoms. Two patients developed chikungunya symptoms after spending at least 2 days in Phuket, two others in Koh Samui, and two others in Koh Lanta. One patient became symptomatic after returning to his country of residence.

These nine confirmed cases demonstrate that travellers are currently at risk of CHIKV infection in many tourist areas in southern Thailand. Only one of them had received pre-travel advice. Furthermore, in September 2018, there was a report to EuroTravnet/GeoSentinel of a suspected chikungunya case in a traveller returning from Malaysia to Spain, and chikungunya cases are currently reported from the Malaysian Ministry of Health [5], indicating an extension of this outbreak to neighbouring Asian countries. Given the global traffic to South East Asia, and Thailand in particular [6], there is a need to raise awareness to travellers and healthcare providers about the risk of arboviral diseases.

As confirmed by local sources [7], Case 4 and her sibling attest that CHIKV and the endemic dengue virus are co-circulating in Thailand. The weekly number of dengue fever cases in Thailand seems similar to those recorded in 2018 at the same period with around 150 cases per week in the South [8]. Furthermore, 15 dengue cases have been recorded in the GeoSentinel database in travellers returned from Thailand since the start of 2019.

Prescription of NSAID for suspected arboviral disease imported from South East Asia should be contraindicated when it is not possible to differentiate between dengue and chikungunya viruses, to avoid haemorrhagic complications. Besides, these are known to be more severe in patients with co-infections [9,10]. In addition, careful attention to adequate hydration is essential to minimise feelings of light-headedness and malaise [4].

A distinguishing characteristic of CHIKV infection, in contrast to dengue, are prolonged musculoskeletal disorders [11]. In this case series, all adult women developed long-lasting joint pains; these symptoms were worst in the oldest case (Case 1) and in Case 9 who was treated early in her disease course with corticosteroids, which are not recommended because of the rebound effect after withdrawal [4]. The management of persistent pains relies mainly on a complete therapeutic dose of NSAID after the acute febrile stage, in combination with physical and rehabilitation medicine [4]. Populations at higher risk of complications, including women older than 40 years, persons with underlying rheumatic diseases and athletes, who are planning travel to Thailand and neighbouring countries, should be made aware of the risk of disabilities [11] and of the need for all-day preventive measures against vectors using physical (clothing, bed nets) and chemical (topical repellents with DEET and permethrin impregnations) barriers [12].

The competent vector *Aedes albopictus* has become widely established in some parts of Europe and Israel since 1979. In 2006, CHIKV was first imported into Europe from outbreaks in the Indian Ocean [13]. Consequently, these locations are at risk of introduction and potential epidemics [14,15].

*Ae. albopictus* can transmit CHIKV strains to a varying degree, depending on the viral genome and environmental temperature [16]. There are three main genotypes of CHIKV: Asian, West African and East/Central/South African (ECSA). The Asian CHIKV, which is more easily transmitted by *Ae. aegypti*, caused the first documented outbreak in Thailand in 1958 [17] and has regularly circulated in South East Asia in recent years. However, it was an ECSA-mutated virus, well adapted to *Ae. albopictus*, that emerged on islands in the Indian Ocean in 2005, spread to Asia and was subsequently responsible for the re-emergence of chikungunya in Thailand in 2008 [18]. Autochthonous transmission events, following ECSA-CHIKV introductions through viraemic travellers, have occurred in Italy in 2007 and 2017 [19] and in France in 2010, 2014 and 2017 [20]. Therefore, determining the CHIKV strain currently circulating in Thailand is of importance to assess the risk of local transmission in Europe, especially before the period of seasonal activity of the vector *Ae. albopictus*.

## Conclusion

These nine cases, occurring over a period of 2 months, indicate a rise in the number of travel-associated CHIKV infections in southern Thailand with a risk of importation to Europe. At the same time, they illustrate the long-term burden of chikungunya infection. Awareness among clinicians, information and appropriate management of travellers returning from Thailand and neighbouring countries, as well as early detection of imported cases and immediate mosquito control strategies, are vital for preventing CHIKV dissemination and related morbidity.
